# A neuroscience perspective on incidental imaging findings and diagnostic and therapeutic silos

**DOI:** 10.3325/cmj.2015.56.68

**Published:** 2015-02

**Authors:** Lukasz M. Konopka

**Affiliations:** Department of Psychiatry, Loyola Medical Center, Maywood Il, USA and Yellowbrick Consultation and Treatment Center, Evanston IL, USA

Traditionally, psychiatry and clinical psychology have understood behavior as separate from brain function and have avoided referring neurobehavioral patients, particularly those carrying a Diagnostic Statistical Manual (DSM) diagnosis, for additional studies such as neuroimaging. Current psychiatric training programs continue to permit this dichotomous misunderstanding by neglecting neuroscience courses that elucidate the relationship between behavior and brain function. Psychiatrists and psychologists have learned to interpret and manage behavior without a clear picture of brain physiology and function (1). Moreover, with the exception of some neuropsychologists, most psychologists and psychiatrists neglect standard neurological exams and only consider behavioral symptoms. When it concerns neurobehavioral disorders, psychiatric and psychological standards of practice disregard objective imaging for assessing brain function and primarily reserve this technique for research. This omission confirms a meager understanding of brain physiology and function and their influences on behavior. Therefore, psychological and psychiatric theories have become stuck in unsubstantiated data. For example, the DSM defines disorders purely from observational data. To be fair, those diagnostic theories evolved during an era that lacked the necessary technology for objective brain evaluation, yet despite neuro-imaging and neuro-scientific advances, clinical psychology and psychiatry training programs still disregard introducing neuroscience to their students trained to be clinicians. Consequently, this omission has caused competition and isolation between clinical specialties and the development of practical and intellectual silos. Didactic programs that ignore neuroscience perspectives hinder clinical mental health students by denying them a holistic view of the patient. This incomplete picture begins the process of pseudo-specialization, a process that has little to do with developing optimal person centered therapeutics for the patient. In an ideal world, all behavioral fields would pursue a common neuroscientific language and education that permits seamless understanding of biology, behavior, sociology, and pathology.

Our current educational and practical standards fuel silos that drive the psychiatric and psychological world and divorce their practices from the clinical and applied neuroscience laboratories that could provide neuro-imaging and neuro-diagnostics – all because they are unaware of the benefits (2). Moreover, when patients need neuroimaging studies, they face major roadblocks. Economic forces have also caused isolation by defining the clinician’s role and standards of practice, eg, insurance providers restrict imaging tools primarily because of costs, but also because they see psychologists as outside the medical model; even psychiatrists can face considerable resistance. Consequently, when patients’ neuro-diagnostic data are neglected or separated from the diagnosis, they end up with seemingly irrational ineffective treatments.

Neuroradiology is another specialty critical for accurate patient diagnosis and treatment, but because radiological images often yield negative structural results, it is also a specialty that often argues against brain imaging for psychiatric patients. Nevertheless, neuroradiologists report negative results because they have used standard practice of subjective visual inspection looking for gross abnormalities such as vascular events, demyelinating disorders, tumors, cysts, and other aberrations (3). This practice continues despite the overwhelming data showing that subtle findings may characterize psychiatric conditions and require statistical analysis for clarification. For example, there are many reports of altered volumetric measures and white matter connectivity that remain undetected by simple visual inspection (4). As such, we should reconsider how we use neuroimaging and engage in more precise statistical evaluations to clarify patient anatomy and how it relates to clinical symptoms. For chronically ill patients, if we cannot perform sophisticated statistical evaluations, we then should still strongly consider standard neuro-imaging with magnetic resonance imaging (MRI).

My personal experience has shown me the importance of incidental imaging findings and makes a strong argument for imaging patients with psychiatric disorders. Our clinic specializes in individuals whose ages range from 18 to 30 and who have suffered prolonged psychiatric illness with multiple hospitalizations and suicide attempts. We evaluate their brain function and behaviors by combining neurobehavioral face-to-face, paper and pencil, and computerized neuropsychological assessments with imaging tools such as quantitative Electroencephalography (EEG) and evoked potentials. Thirteen patients whose psychiatric histories and neurobehavioral findings were significantly abnormal received a combination of MRI and positron emission tomography (PET)-EEG. Although a surprising number showed structural abnormalities, from their clinical histories and neurobehavioral assessments their specific findings would have been totally unpredictable. Their only shared clinical characteristics were significant difficulty in school combined with refractory psychiatric symptoms. Surprisingly, 3 out of the 13 had arachnoid cysts in the posterior fossae. So, despite begin considered incidental, the frequency of the cysts was actually quite extraordinary! In the general population, cysts occur in 5 cases out of 1000 examined (5), and 22 cysts occurred in 2000 studies (6). Obviously our hit-rate was much higher. It is important to note that we did not refer these patients for imaging because of their significant psychiatric, cognitive, or emotional challenges but, primarily, because of their quantitative EEG findings (7). Therefore, patients with difficult developmental histories and neurobehavioral symptoms that resist psychiatric or psychological interventions should be fully evaluated as soon as possible with all available objective tools. Yet, the question remains how such findings impact a patient’s clinical course. Why these cysts developed was unclear, but undoubtedly, had our patients discovered their cysts in early childhood and had they been monitored, their cysts may have been less likely to enlarge, treated more easily, and, perhaps, might not have impacted their future psychological development and behavior. Significantly altered perspectives emerged after we presented identifiable brain findings to the patients, their families, and their clinicians. The new data resulted in fresh therapeutic approaches, reevaluated and readjusted attitudes, and elicited completely novel, unique conceptualizations of the case (8). When the family discovered the data, it was an explanation that helped change how they viewed their experiences with and attitudes toward the patient. The discovery altered their compassion, frustration, shame, blame, not to mention their hope in potential subsequent neurosurgical and neurological interventions.

In summary, our understanding of brain function and how it relates to structure and specific neurophysiological patterns is still in its infancy. By dissolving specialty silos and opening communication among professionals, who all treat the same organ – the brain, we will enhance our understanding of pathology and develop multidisciplinary treatments that integrate all available data into clear conceptualizations of each patient. Moreover, the converging data from objective outcome assessments could aid the development of new theories, the modification of original theories, decrease the cost of care, and, ultimately, reduce the burden of mental illness on society, families, and patients.
